# Analyzing gender differentials in dietary diversity across urban and peri-urban areas of Hyderabad, India

**DOI:** 10.1186/s40795-023-00692-2

**Published:** 2023-02-27

**Authors:** Kiran Suryasai Marla, Ravula Padmaja

**Affiliations:** 1 Borlaug-Ruan Intern (2021) at International Crops Research Institute for the Semi-Arid Tropics (ICRISAT), Patancheru, Telangana 502324 India; 2 University of California Los Angeles, 405 Hilgard Avenue, Los Angeles, CA 90095 USA; 3 Sociologist, Gender and Nutrition Research, International Crops Research Institute for the Semi-Arid Tropics (ICRISAT), Patancheru, Telangana 502324 India

**Keywords:** Dietary diversity, Gender disparity, India, Urbanization, Food security, Women

## Abstract

**Background:**

India’s recent increase in urbanization alongside with feminization of rural agriculture could increase the existing gender disparities in dietary diversity. With many rural men migrating to urban areas, women have increased domestic burdens as well as productive burdens such as making informed crop production decisions so household members consume a diverse diet. Given the rapid and recent onset of this phenomenon, there is a need to explore gender differentials in diet diversity across urban and rural areas to assess if certain populations are being disproportionately impacted by this trend. There are limited established quantitative studies discussing this gender disparity with respect to urbanization. Therefore, this paper compares dietary diversity among adult men, adult women, adolescent males, and adolescent females in urban and peri-urban locations. The authors also assess if various sociodemographic factors correlate with dietary diversity.

**Methods:**

Analyses were conducted on dietary diversity data collected by the International Crops Research Institute for the Semi-Arid Tropics (ICRISAT) from selected urban (1108 individuals) and peri-urban (808 individuals) locations of Hyderabad, India. The total sample size of the population is *n* = 1816: 660 adult males, 662 adult females, 205 adolescent males, and 289 adolescent females.

**Results:**

Adult women and adolescent females have a higher diet disparity between peri-urban and urban areas when compared to adult males and adolescent males. Multivariate analyses followed by post hoc multiple comparisons testing further support that peri-urban adult women consume a less diverse diet compared to their urban counterparts and less than other peri-urban adult men and adolescent women. It was also found that marital status, type of household card owned, and the highest degree of education are statistically significant correlators of an individual’s dietary diversity.

**Conclusions:**

Given that urbanization could negatively impact already vulnerable populations such as peri-urban adult women, who play a key role in children’s nutrition, it is important to provide support to these populations. This paper suggests it is possible to do so through government subsidization of peri-urban farmers to grow more diverse crops, fortifying easily accessible foods with commonly lacking micronutrients, including Vitamin A, folic acid, and iron, market access, and affordable prices.

**Supplementary Information:**

The online version contains supplementary material available at 10.1186/s40795-023-00692-2.

## Background

Food insecurity is particularly high in India, as it is ranked 80 out of 104 countries on the Global Hunger Index. Furthermore, India is 130 out of 155 countries on the Gender Inequality Index, and it is clear there are existing gender disparities in food insecurity [1]. Approximately half of the women of reproductive age have anemia, resulting from iron deficiency, while this issue is prevalent in only 23% of men [2]. Malnutrition in women is important to focus on because inadequate health of the mother can lead to inadequate nutrition of a baby, which is correlated with childhood/adolescent stunting and other life-altering side effects such as the increased risk of adult chronic disease [3].

Dietary diversity is a useful measure of one’s food security. Dietary diversity corresponds to a 0.7% increase in per capita caloric availability [4]. Therefore, this measure can be used to both assess energy intake along with micronutrient levels. Using dietary diversity to quantify food insecurity allows researchers to explore the different factors that may contribute to discrepancies in nutritional access across various populations.

Urbanization has not been researched in-depth with respect to its impacts on food security. India’s rate of urbanization recently increased to 2.33% [5]. This rapid increase in rate could lead to disparities in food access across urban and rural areas. Current literature suggests a gendered disparity can be partly attributed to India’s feminization of agriculture, a trend prevalent in many developing countries. While many young men are moving from rural to urban areas, rural women are continuing to do domestic work along with having the role of the temporary household head [6]. Most rural households manage farms jointly, with women in charge of post-harvesting duties through family labor such as cooking and men in charge of crop production labor as well as crop production decision-making [7]. The additional responsibilities for women due to rural men leaving include increased physical labor and ensuring that agricultural decisions are made such that they are still consuming a nutritionally adequate diet [8]. There exist qualitative studies comparing these food systems. For instance, it was found that rural female farmers have more difficulty accessing resources needed for agriculture compared to male farmers [9]. However, there is limited literature quantifying gender disparities in diet diversity across urban and peri-urban areas, particularly in the context of rapid urbanization and the feminization of agriculture.

This study investigates differentials in dietary diversity across urban and peri-urban areas in Telangana, India between adult males, adult females, adolescent males, and adolescent females. The paper also explores other possible factors that could be used as indicators of diet diversity, including marital status, whether an individual attended school, their highest degree of education, and the type of household card. The authors hypothesize that adult women residing in peri-urban locations in Hyderabad are consuming less diverse diets compared to other urban adult women and compared to other peri-urban adult males. We also hypothesize that marital status, highest educational degree received, and BMI range are relevant indicators of dietary diversity, as these sociodemographic factors are statistically correlated with an individual’s dietary diversity score.

## Methods

**Fig. 1:**
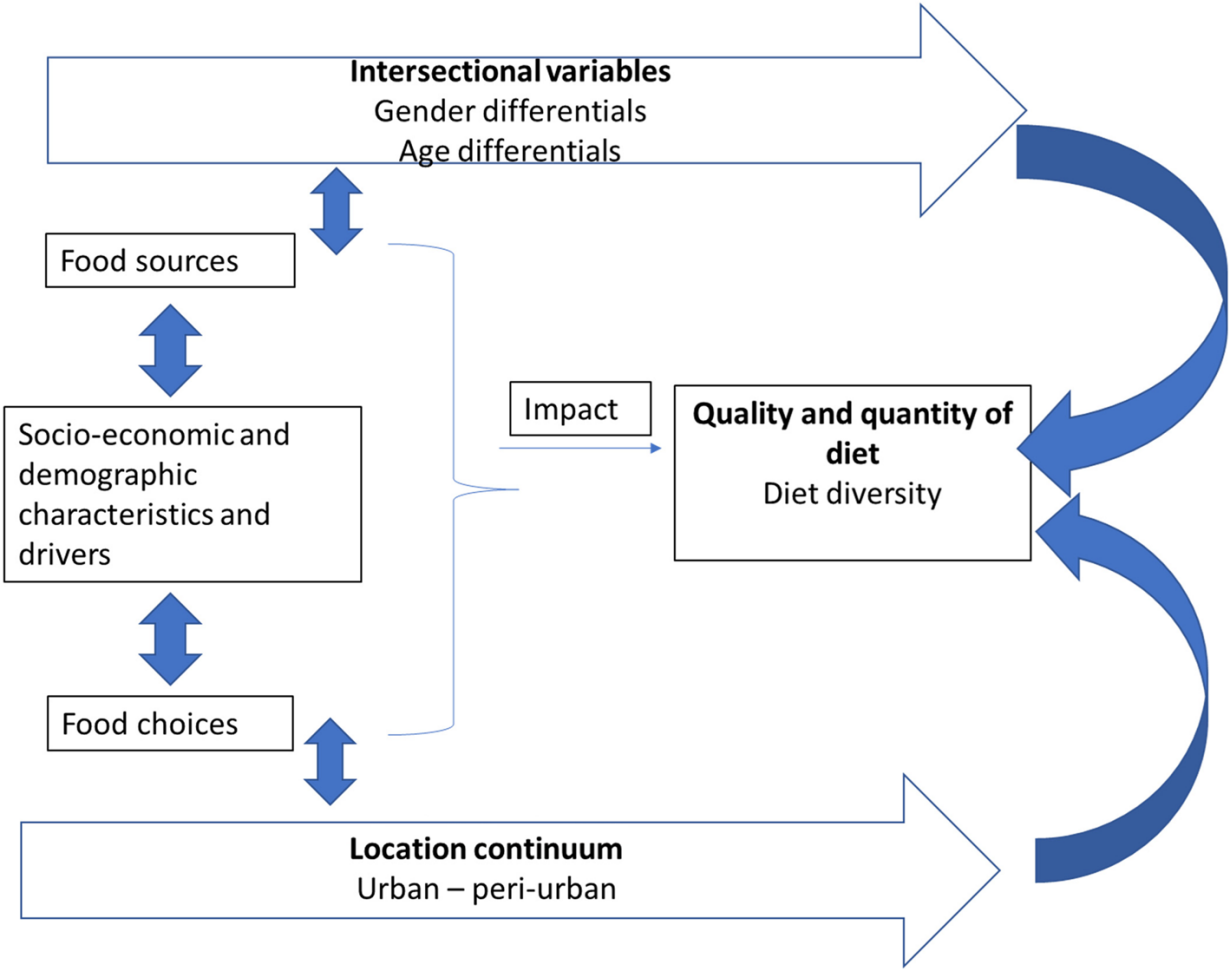
Conceptual framework showing linkages between the independent variables-location and gender with the dependent variable-diet diversity

Figure 1 above details the conceptual framework for this paper. This study uses location and member group as two independent categorical variables to support that peri-urban adult women are consuming a less diverse diet. Individuals’ locations were categorized as either urban or peri-urban and their member groups as adult male, adult females, adolescent males, or adolescent females. Member group includes both gender and age differentials, as shown above. The four sociodemographic variables that were explored as potential indicators of dietary diversity are highest educational degree, type of household card (a measure of socioeconomic status), BMI range, and marital status. Levels within each variable are detailed below in Table 1.

**Table 1:** Variables for multivariate regression

Highest educational degree	Type of household card	BMI range	Marital status
No school	BPL - Below Poverty Line	Underweight - BMI < 18.5	Married
Primary level (classes 1-5)	APL - Above Poverty Line	Normal - 18.5 ≤ BMI < 23	Single/Never married
Secondary level (classes 6-10)	Other - No Ration Card	Overweight - 23 ≤ BMI < 25	Widowed
Technical or vocational training		Obese - BMI ≥ 25	
Intermediate			
Graduate /University (B Sc/BA/B Com/B Tech)			
Postgraduate			
Other			

### Data collection

Data was collected by other team members based on guidelines described in the Urban Sprawl Project Data Collection Documentation report [10]. Individual participants from randomly selected households were surveyed on their consumption of food groups based on a 24-hour recall basis along with sociodemographic information. Other information that was collected can be found in the Urban Sprawl Documentation report. Not all factors were included in the analysis due to a small sample size or lack of relevance. Refer to Supplementary Tables 1 and 2 in Appendix A [see Additional file 1] for other information collected. Within each household, three to four members were selected such that there were an adult male and female (20-65 years), adolescents (11-15 years), and children (3-5 years). Four sites in Hyderabad, India were chosen for data collection, two of which are urban areas and the other two peri-urban. Table 2 reflects household information of locations surveyed. Data was collected every 6 months three separate times. This project explores the data from the first round of data collection.

**Table 2:** Locations of households surveyed

Location	Urban/Peri-urban	Number of households
Afzalgunj	Urban	55
Ramachandrapuram	Peri-urban	61
Sri Sai nagar (Bachupally)	Peri-urban	57
Begumpet	Urban	45
Chandrayangutta	Urban	46
Langerguda/Gaganpahad	Urban	45
Thimmaiguda (Gowraram)	Peri-urban	35
KPHB Phase7	Peri-urban	60
Lingampally	Peri-urban	60
Madhapur	Urban	45
Mallapur	Peri-urban	31
Sai nagar (Nagole)	Peri-urban	35
Pocharam	Peri-urban	32
Tolichowki	Urban	55
Total Number of Households		662

### Dietary diversity scores (DDS)

Dietary diversity scores were computed based on surveys conducted by trained enumerators. The FAO details ten food groups reflecting micronutrient adequacy for women that were used in this paper to indicate the dietary diversity level of individual adult males, adult females, adolescent males, and adolescent females. Consumption of these food groups is quantified through Minimum Dietary Diversity for Women (MDD-W). This metric is recommended to be used for adult women and adolescent girls of reproductive age [11]. Given the lack of a validated metric to assess dietary diversity for men, the authors used MDD-W to assess and compare the adequacy of diet for adult females, adult females, adolescent males, and adolescent females. This type of assessment was replicated in other research studies. An example involves a paper that also used MDD-W to develop an association between intra-household dietary diversity and crop and income diversity among men and women in the same household [12]. Another study comparing men’s and women’s diets living in the rural-urban interface also used the same DDS metric for their quantitative analysis [13]. MDD-W cannot be used to assess children’s diet diversity, as another metric is used for this analysis. Therefore, children are not included in this dataset. The total sample size of the population is *n* = 1816: 660 adult males, 662 adult females, 205 adolescent males, and 289 adolescent females. One thousand one hundred eight individuals lived in peri-urban areas, and 808 individuals lived in urban areas. Sample sizes for each member group and location are listed below in Table 3.

**Table 3:** Two-way table of members groups across urban and peri-urban regions

	Peri-urban	Urban	Total
Adult male	369	291	660
Adult female	371	291	662
Adolescent male	120	85	205
Adolescent female	148	141	289
Total	1008	808	1816

Values are sample sizes

The ten food groups underlying MDD-W are 1) Grains, white roots and tubers, and plantains 2) Pulses (beans, peas, and lentils) 3) Nuts and seeds 4) Dairy 5) Meat, poultry, and fish 6) Eggs 7) Dark green leafy vegetables 8) Other vitamin A-rich fruits and vegetables 9) Other vegetables 10) Other fruits. Consuming less than 50% of food groups, or 5 groups, is defined as below minimally adequate diet diversity [11]. Raw data including all dietary diversity information collected is provided in Additional file 2 as an excel sheet.

### Data analysis

The authors analyzed data collected by enumerators via R, Jamovi, and Microsoft Excel. Based on the consumption of each food group, dietary diversity scores were computed. If consumed, a score of 1 was assigned to the individual for the corresponding food group. If it was not consumed, then a score of 0 was assigned. Total dietary diversity scores were computed by adding food consumption scores for each food group. Computed scores were then validated by confirming that DDS scores were between 0 and 10.

Microsoft excel sheets with collected data were imported to RStudio for data cleaning and transposing using the “dplyr” and “tidyverse” packages in RStudio so that all indicators of interest (ex. member group, location, marital status, etc.) were available in one data frame. Then, the data frame was uploaded to Jamovi, a free open-source software. Jamovi was also used to create Descriptive tables, which include count, mean, standard deviation, maximum, and minimum. After identifying possible indicators of DDS differentials among participants in Jamovi, the data frame was then analyzed in RStudio to conduct more complex analyses.

A questionnaire was also generated via Google Forms to assess the food environment of a literate population in Patancheru, separate from the surveyed members in this study. This was used to assess the frequency of food group consumption, food accessibility, and attitudes toward nutritional health. The data collected through this survey was not included in this report.

### Statistical model

The authors provided a histogram to visualize the consumption of food groups across various member groups. The authors then tabulated a comparison of the proportion of member groups living in urban and peri-urban areas who are below a minimally adequate diet diversity level (DDS < 5). A fisher exact test was conducted to assess if differences in proportions across urban and rural areas were significant for each member group. An alpha significance of 0.05 was used. Other papers used a similar analysis method to compare proportions of populations consuming an adequate diet [14–16].

**Fig. 2:**
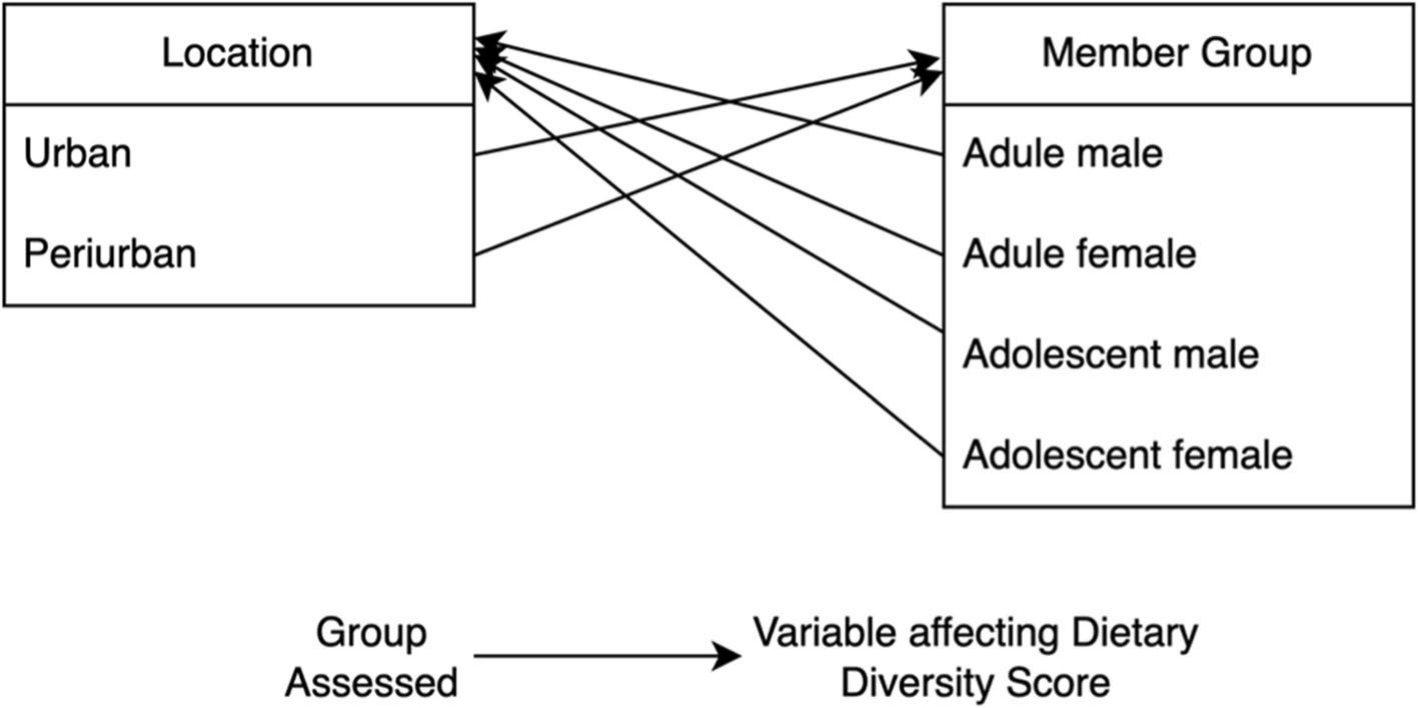
Factorial design of ANOVA

To fully establish the significance and effect of disparities among member groups and locations, a multivariate analysis was conducted to develop a bivariate association across the two independent variables. As shown in Fig. 2, these two variables are assessed together using a factorial design; each member group (4 groups) was tested to assess if the location makes a significant impact on DDS, and individuals living in each location (2 locations) were tested to assess if the member group they identify as makes a significant impact on DDS.

The mean differences in DDS between two groups were compared via an independent sample t-test, while more than two means were compared via one-way analysis of variance (ANOVA). When conducting repeated sample t-tests, Bonferroni’s multiple testing correction was applied to develop an adjusted alpha-value by dividing the initial alpha value (0.05) by the number of comparisons made to output the corrected α. ANOVA tests were reported with post-hoc Tukey HSD tests to identify which member groups were statistically impacted by which location, and vice versa. Results outputted an adjusted *p*-value so α = 0.05 was used.

After running statistical analyses to support the hypothesis that adult females are being disproportionately impacted in peri-urban areas, multivariate ordinary least squares regression was conducted on other relevant socio-demographic information collected: highest educational degree, type of household card, BMI range, and marital status. Each independent variable was used to formulate correlation coefficients with the dependent variable DDS. A *p*-value of less than 0.05 is defined as statistically significant for the model as a whole and levels within each independent variable.

## Results

**Fig. 3:**
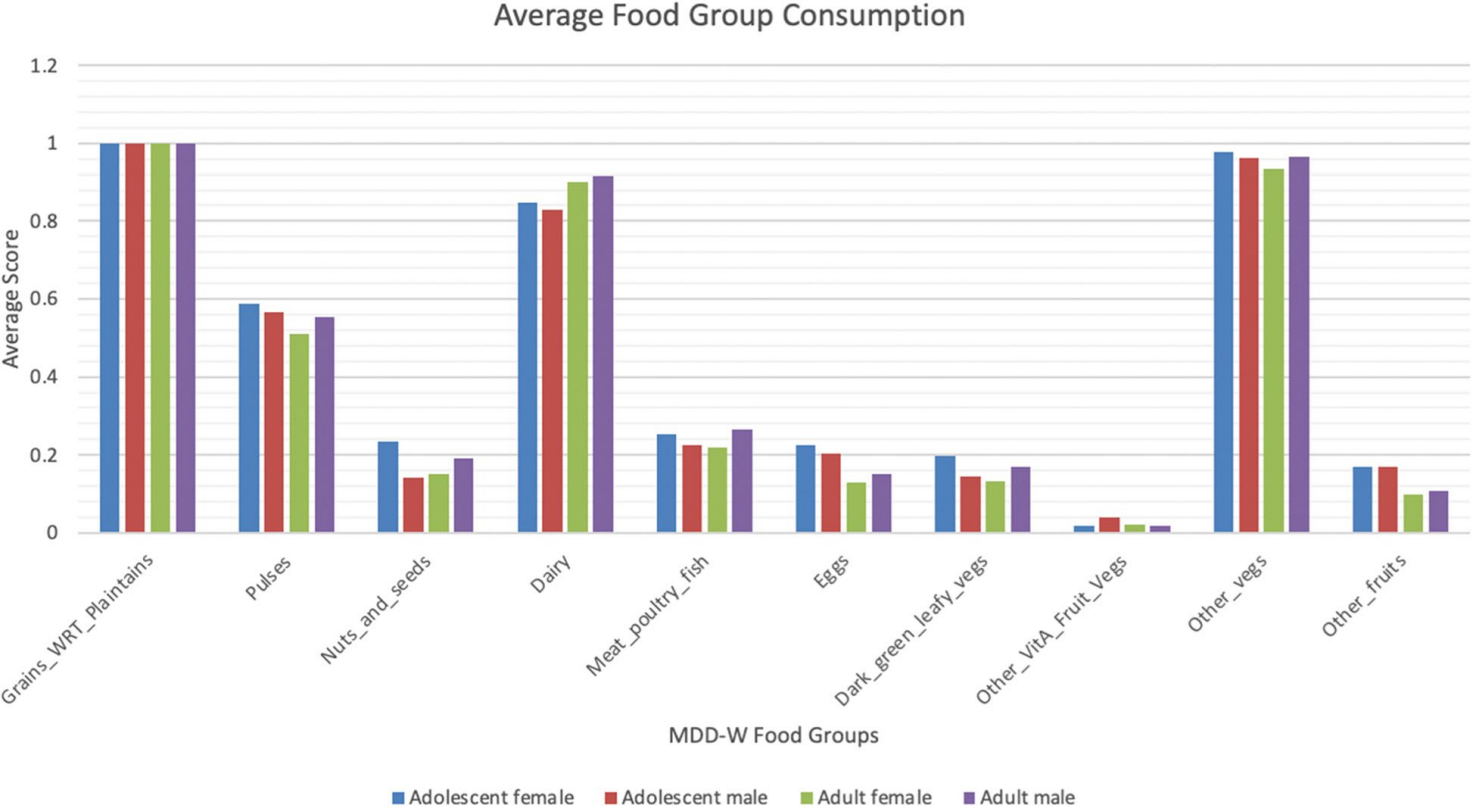
Histogram of average food group score of each member group

After the initial analysis was done on the consumption of each of the food groups consumed by the different member groups in a household, Fig. 3 indicates that the top three most consumed food groups were food groups 1 (Grains, white root tubers, and plantains), 4 (dairy), and 9 (other vegetables). The least consumed groups were food groups 7 (dark green leafy vegetables), 10 (other fruits), and 8 (other vitamin A-rich fruits and vegetables). A descriptive table breakdown of average food group consumption by each member group is provided in Supplementary Table 3 in Appendix B [see Additional file 1]. A score of 1 is defined as an individual consuming the food group, while 0 is defined as a lack of consumption.

### Diet diversity in urban and peri-urban areas - a gendered analysis

Minimum diet diversity, which is defined as consuming below 50% of total food groups on a given day, was compared across member groups and locations in Table 4. The results of the fisher exact test indicate that adult females had a significant disparity in their proportion of population below minimum diet quality when comparing urban and peri-urban counterparts (*p* < 0.001). All other member groups do not have a significant difference in proportion (*p* > 0.05).

**Table 4:** Fisher’s exact test results of population proportions below minimum diet diversity

Member group	Peri-urban	Urban	*P*-value
Adult male	60.976	57.732	0.425
Adult female	65.368	57.732	** < 0.001
Adolescent male	56.667	60.000	0.668
Adolescent female	57.432	49.645	0.196

Values are population percentages below a minimum dietary diversity (MDD < 5)

**p < 0.001

An analysis of variance was then conducted to assess how location impacts each of the different member group’s (adult male, adult female, adolescent male, adolescent female) DDS and how the DDS of individuals living in each of the two locations (urban and peri-urban) are impacted by identifying as a particular member group. This factorial design is visualized in the methods section above in Fig. 2. Table 5 indicates the results of the multivariate analysis. Bonferroni’s multiple testing correction was applied. Across the four member groups, there were four independent sample t-tests conducted comparing DDS of those living in peri-urban and urban areas. Therefore, the adjusted cutoff for significance was α = 0.0125. Adjusted *p*-values were used for Tukey HSD tests when comparing more than two means.

**Table 5:** Analysis of variance of DDS with post hoc Tukey HSD results

		Dietary diversity scores			
Characteristic	Category	Mean	SD	Minimum	Maximum
**Member Group**					
Adult male	Peri-urban	4.330	1.184	2	8
	Urban	4.347	1.184	1	8
Adult female	Peri-urban	4.100*	1.207	2	8
	Urban	4.347	1.174	2	8
Adolescent male	Peri-urban	4.292	1.331	2	8
	Urban	4.271	1.189	2	7
Adolescent female	Peri-urban	4.520	1.343	2	9
	Urban	4.504	1.371	2	8
**Location**					
Urban	Adult male	4.347^a^	1.184	1	8
	Adult female	4.347^a^	1.174	2	8
	Adolescent male	4.271^a^	1.189	2	7
	Adolescent female	4.504^a^	1.371	2	8
Peri-urban	Adult male	4.330^a^	1.184	2	8
	Adult female	4.100^b^	1.207	2	8
	Adolescent male	4.292^a,b^	1.331	2	8
	Adolescent female	4.520^a^	1.343	2	9

^*^Mean values were significantly different as determined by independent t-tests (*p* < 0.0125)

^a,b^Mean values with different superscript letters were significantly different as determined by ANOVA with post hoc Tukey HSD test (adjusted *p* < 0.05)

Based on the results, only adult females’ DDS are statistically impacted by their location, with peri-urban members having a lower dietary diversity; Peri-urban and urban adult women had different mean DDS of 4.100 and 4.347, respectively, with a significant p-value. Subscripts indicate specific pairwise comparisons of DDS between member groups living in rural and urban areas. Mean DDS of all member groups residing in urban areas are statistically similar, while this is not the case for those living in peri-urban areas; Peri-urban adult women (Mean DDS = 4.100) have a statistically different (adjusted *p* < 0.05) dietary diversity than peri-urban adult males (Mean DDS = 4.330) and adolescent females (Mean DDS =4.520). Adult women residing in peri-urban areas again have lower mean DDS. Urban adult women have statistically similar DDS compared to other urban member groups.

### Exploring indicators of dietary diversity

Multivariable regression was conducted to assess the value of other socio-demographic variables as potential correlators of dietary diversity. The variables assessed in this model were BMI range, type of household card received, marital status, and highest educational degree earned (Table 6). Not all levels listed in Table 1 were included in the regression due to a low sample size (*n* ≤ 10). A full descriptive breakdown of sample size, mean DDS, and SD of each sociodemographic variable analyzed can be found in Supplementary Table 4.

**Table 6:** Association between the socio-demographic variables and DDS

	β Coefficients	Standard error	***P***-value
**BMI Range**			
Underweight	Reference		
Normal	0.166	0.102	0.105
Overweight	0.245	0.133	0.066*
Obese	0.400	0.110	< 0.001**
**Type of Household Card**			
Other - No Ration Card	Reference		
BPL	0.156	0.105	0.138
APL	0.458	0.169	0.007*
**Marital Status**			
Single/Never Married	Reference		
Married	−0.315	0.097	0.001*
**Highest Educational Degree**			
Primary Level	Reference		
Secondary Level	−0.068	0.083	0.409
Intermediate	−0.027	0.138	0.844
Graduate/University	0.342	0.151	0.024*
Postgraduate	0.122	0.224	0.586
Adjusted F	3.236		
*P*-value	< 0.001**		

* *p* < 0.05

** *p* < 0.001

Regression results indicate that BMI, type of household card, and highest educational degree are positively associated with dietary diversity. Those with a higher BMI relative to the underweight group have a higher dietary diversity score (β = 0.235, SE = 0.133 for overweight and β = 0.400, SE = 0.110 for obese). Individuals holding a governmental APL household card consume a more diverse than those without access to a ration card (β = 0.458, SE = 0.169). Compared to single/never married participants, married participants have a lower diet diversity (β = − 0.315, SE = 0.097). Members who completed a graduate/university level education consume a more diverse diet than those who have only completed a primary level of education (β = 0.342, SE = 0.151). The overall regression model has a *p*-value less than 0.001 (F = 3.236).

## Discussion

The results presented in the paper indicate that while every participant consumed Food Group 1, only about 15% consumed dark green leafy vegetables, other fruits, and other Vitamin A-rich fruits and vegetables. A lack of these food groups can lead to adverse health effects, including vision loss, skin issues, and immune system deficiencies [17].

These results are also corroborated by health initiatives that have taken place in India. The government implemented a Vitamin-A supplementation policy in response to the large healthcare burden India’s system is facing due to this deficiency, particularly nutritional blindness from Vitamin A. This key nutrient is mainly found in all three of the least consumed food groups from the population studied. In fact, many of those living in South India also face additional multiple micronutrient deficiencies in folic acid and Vitamin C, which are also all generally consumed in the diet through the three lowest consumed food groups [18].

These findings are also consistent with focus group discussions conducted by the enumerators, as many participants mentioned that dark green leafy vegetables and fruit were particularly expensive and had significantly increased in cost during the COVID-19 pandemic. Another study focusing on diets during the COVID-19 pandemic further corroborated these findings, as it was found that women and households altogether have been spending less on non-staples, which include non-grain foods. The study discusses a shift away from vegetables and meats towards more affordable staples such as cereals, which is seen from the 100% consumption in food group 1, due to the “disproportionate increase in nonstaples prices compared to staple foods.” [19].

The results of the multivariate analysis specifically quantified the gender disparity by assessing differences in DDS between member groups and locations. Post-hoc multiple testing via pairwise Tukey t-test further supported the hypothesis because it indicates two layers of disparity among adult females; they are the only member group to have a locational disparity across urban and peri-urban areas, with peri-urban adult females consuming a less diverse diet. And within peri-urban areas, adult females are consuming a less diverse diet than their adult male counterparts. As mentioned, there are limited research studies assessing bivariate associations of assessing member groups and location, specifically with respect to urbanization and dietary diversity. However, a study researching diet diversity between women and men found a similar trend; peri-urban populations had a lower dietary diversity score compared to their urban counterparts. Particularly, women living in peri-urban and peri-urban areas had the unhealthiest food consumption and lowest diet diversity, while this was not true for men [18]. Other studies that have rural-urban or gendered comparisons in dietary diversity have followed a similar analysis method of ANOVA with post-hoc Tukey tests [20–22]. Other researchers assessing rural obesity found statistically significant associations between rising obesity in India and the urbanization of its rural spaces [23]. A third study also researching urban household food security in South Africa concluded that there was a lower average number of micronutrients consumed in rural areas and this disparity is closely associated with increased urbanization. The authors generalized this increasing disparity to other developing countries facing similar geopolitical trends, such as India [24]. Although the paper did not assess gender disparity and only focused on locational disparity, the results of this study alongside existing limited literature support the need to provide a support system for adult women within peri-urban areas; this population particularly needs the resources to control their agricultural output since many men are rapidly leaving peri-urban areas along with their previous farming responsibilities [25].

The regression suggests the sociodemographic factors marital status, highest educational degree, BMI range, and type of household card are statistically significant variables that impact dietary diversity. Findings are also supported by several other research studies whose analyses indicate these factors are correlated with DDS [26]. Although the four variables have statistically notable impacts on DDS, the multivariable linear regression model as a whole cannot be used to accurately predict dietary diversity scores. Diet quality is impacted by a multitude of biological, social, economic, and psychological factors, some of which include appetite, genetic predispositions, mood, stress, and culture. It is not possible to incorporate all factors that accurately account for the variance in an individual’s DDS [27]. Nonetheless, it is crucial to identify the factors that significantly impact one’s diet quality for a given population.

There are several limitations to our paper. First, the authors used MDD-W to assess the dietary diversity of adult and adolescent males because there are not any standardized measures to assess the dietary diversity of men. Other researchers have also been using this metric to compare these member groups’ dietary diversity. However, future research could analyze the validity of comparing men’s and women’s diet quality with the ten food groups detailed in this study. Second, this paper reports the results of a cross-sectional study. Although the analysis with the literature review provides correlation conclusions, this research design cannot be used to establish a cause-and-effect relationship between urbanization and increasing gender disparities in dietary diversity. We suggest that future research analyzes the long-term effects of urbanization by following the same community members for several years and collecting dietary data. Third, there was a low sample size across several levels within various sociodemographic variables such as marital status, and educational degree received. A large sample dataset (*n* > 1800) was aggregated to account for several levels. However, to provide comprehensive pairwise comparisons between categories within each variable, more data should be systematically collected on individuals with these characteristics.

## Conclusions

Based on the data collected and analysis conducted, there is a lack of consumption of dark green leafy vegetables and vitamin A-rich fruit and vegetables, which could lead to micronutrient deficiencies and adverse health effects, an already prevalent issue in this area of India. Since every member in the study had consumed grains, white root tubers, and plantains on a given day, it could be beneficial to implement fortification of these staples with Vitamin A, folic acid, iron, and other micronutrients individuals are lacking due to low consumption of these food groups. Fortifying easily accessible foods such as grains and white root tubers would allow community members to consume these important nutrients while mitigating financial and accessibility constraints. GAIN (Global Alliance for Improved Nutrition) already has existing programs and partnerships with India such as the implementation of large-scale food fortification and distribution of better-quality food to children in school [28]. The development of a program through GAIN to help specifically address the fortification of peri-urban Indian communities could help mitigate the negative dietary impacts of urbanization. Given that GAIN already has a partnership with India and has a mission focused on addressing diet, this intervention could be effective.

The authors also suggest that the Indian government supports peri-urban farmers, particularly women, who locally grow and sell crops containing under-consumed food groups such as dark green leafy vegetables and other Vitamin A-rich foods. A significant reason participants stated they were not consuming these food groups was the lack of affordability. Subsidizing a portion of farming costs would incentivize farmers to grow more of these crops and sell them at a lower cost, which could increase accessibility for peri-urban community members.

There are significant disparities in diet diversity between peri-urban and urban women/adolescent females, whose population percentage under a minimum diet diversity is already significantly higher compared to men. Therefore, it is crucial to assure that the undernourished in peri-urban areas, specifically women and adolescent females, are not left behind during this mass migration to urban areas. After synthesizing the conclusions of other researchers studying this topic with the results of this paper, the authors conclude that developing programs with existing partnerships/alliances as well as government subsidization of farmers can help counteract the negative impacts of urbanization and ensure resources for these vulnerable populations to access a healthy, nutritious diet for generations moving forward [20, 29, 30].

## Supplementary Information

**Additional file 1: Appendix A.** Nutritional Status Information Collected. **Supplementary Table 1.** Anthropometric Readings Collected. **Supplementary Table 2.** Morbidity Patterns Collected. **Appendix B.** Descriptive Tables. **Supplementary Table 3.** Descriptive Table of Food Group Consumption by Member Group. **Supplementary Table 4.** Descriptive Table of Sociodemographic Variable Levels.

**Additional file 2: **Raw Dietary Diversity Data Collected. Raw Dietary Diversity Data Collected. Excel sheet of food consumption data collected from participants by enumerators.

## Data Availability

All data generated or analyzed during this study are included in this published article and its supplementary information files.

## Abbreviations

ICRISAT: International Crops Research Institute for the Semi-Arid Tropics
MDD-W: Minimum Dietary Diversity for Women
DDS: Dietary Diversity Score
ANOVA: One Way Analysis of Variance
HSD: Honestly Significant Difference
SD: Standard Deviation
SE: Standard Error

## References

[1] PIB Delhi. Global Gender Gap Report. 2021. https://pib.gov.in/pib.gov.in/Pressreleaseshare.aspx?PRID=1782628.

[2] Sedlander E, Talegawkar S, Ganjoo R, Ladwa C, DiPietro L, Aluc A, Rimal RN (2021). How gender norms affect anemia in select villages in rural Odisha, India: a qualitative study. Nutrition.

[3] Elder L, Ransom E. Nutrition of women and adolescent girls: why it matters: PRB; 2003. https://www.prb.org/resources/nutrition-of-women-and-adolescent-girls-why-it-matters/

[4] Ruel MT (2003). Operationalizing dietary diversity: a review of measurement issues and research priorities. J Nutr.

[5] Central Intelligence Agency. The World Factbook. India; 2021. https://www.cia.gov/the-world-factbook/countries/india/.

[6] Das A, Mohapatra S, Patnaik NM (2021). Feminization of Indian agriculture: a review. Agric Rev.

[7] Krishna VV, Veettil PC (2022). Gender, caste, and heterogeneous farmer preferences for wheat varietal traits in rural India. PLoS One.

[8] Pattnaik I, Lahiri-Dutt K, Lockie S, Pritchard B (2018). The feminization of agriculture or the feminization of agrarian distress? Tracking the trajectory of women in agriculture in India. J Asia Pac Econ.

[9] Argaw TL, Phimister E, Roberts D (2021). From farm to kitchen: how gender affects production diversity and the dietary intake of farm households in Ethiopia. J Agric Econ.

[10] Padmaja R, Nedumaran S, Lagerkvist C, Kasala K, Kumar R (2018). Documentation of data collection urban sprawl project in Telangana, India 2018-2020.

[11] FAO, USAID, & FANTA III (2016). Minimum dietary diversity for women- a guide to measurement.

[12] Singh S, Jones AD, DeFries RS, Jain M (2020). The association between crop and income diversity and farmer intra-household dietary diversity in India. Food Secur.

[13] Geetha K, Yatnatti S, Vijayalakshmi D, Dittrich C. Food consumption practices of men and women across rural-urban Interface of south Indian megacity Bangalore. Eur J Nutr Food Saf. 2020:1–9. 10.9734/ejnfs/2020/v12i530223.

[14] Hasan M, Islam MM, Mubarak E, Haque MA, Choudhury N, Ahmed T (2018). Mother’s dietary diversity and association with stunting among children <2 years old in a low socio-economic environment: a case–control study in an urban care setting in Dhaka, Bangladesh. Matern Child Nutr.

[15] Kornatowski BM, Comstock SS (2018). Dietary diversity is inversely correlated with pre-pregnancy body mass index among women in a Michigan pregnancy cohort. PeerJ.

[16] Zhong W, Zhao A, Lan H, Mao S, Li P, Jiang H, Wang P, Szeto IM-Y, Zhang Y (2022). Dietary diversity, micronutrient adequacy and bone status during pregnancy: a study in urban China from 2019 to 2020. Nutrients.

[17] Cleveland Clinic. Vitamin a deficiency: causes, symptoms, treatment & prevention: Cleveland Clinic; 2022. https://my.clevelandclinic.org/health/diseases/23107-vitamin-a-deficiency.

[18] Gonmei Z, Toteja GS (2018). Micronutrient status of Indian population. Indian J Med Res.

[19] Gupta S, Seth P, Abraham M, Pingali P. COVID-19 and women’s nutrition security: panel data evidence from rural India. Econ Polit. 2021. 10.1007/s40888-021-00233-9. 10.1007/s40888-021-00233-9 PMC8249434 35422584

[20] Nguyen PH, Scott S, Headey D, Singh N, Tran LM, Menon P, Ruel MT (2021). The double burden of malnutrition in India: trends and inequalities (2006–2016). PLoS One.

[21] Shaun MMA, Nizum MWR, Shuvo MA, Fayeza F, Faruk MO, Alam MF, Hawlader MDH, Mali SK (2023). Determinants of minimum dietary diversity of lactating mothers in rural northern region of Bangladesh: a community-based cross-sectional study. Heliyon.

[22] Worku T, Gonete KA, Muhammad EA, Atnafu A (2020). Sustainable under nutrition reduction program and dietary diversity among children’s aged 6–23 months, Northwest Ethiopia: comparative cross-sectional study. Int J Equity Health.

[23] Aiyar A, Rahman A, Pingali P (2021). India’s rural transformation and rising obesity burden. World Dev.

[24] Ebenezer M (2022). Urban household food security: an assessment of the correlates of micronutrient-sensitive dietary diversity. J Dev Areas.

[25] Jonah CMP, May JD (2020). The nexus between urbanization and food insecurity in South Africa: does the type of dwelling matter?. Int J Urban Sustain Dev.

[26] Claudia G-C, Lucia M-S, Miguel K-K, Patricia C, Edgar D-G (2019). Association between sociodemographic factors and dietary patterns in children under 24 months of age: a systematic review. Nutrients.

[27] Jackson RT. Some factors influencing variation in nutritional needs and requirements of children. J Childrens Health. 2011. 10.3109/713610279.

[28] GAIN. Global Alliance for improved nutrition (GAIN). India; n.d. 2023. Retrieved Dec 31, 2021, from https://www.gainhealth.org/impact/countries/india.

[29] Vilar-Compte M, Burrola-Méndez S, Lozano-Marrufo A, Ferré-Eguiluz I, Flores D, Gaitán-Rossi P, Teruel G, Pérez-Escamilla R (2021). Urban poverty and nutrition challenges associated with accessibility to a healthy diet: a global systematic literature review. Int J Equity Health.

[30] Amare M, Arndt C, Abay KA, Benson T (2020). Urbanization and child nutritional outcomes. World Bank Econ Rev.

