# Supplementary material for: Analyzing gender differentials in dietary diversity across urban and peri-urban areas of Hyderabad, India
Source: BMC Nutr. 2023 Feb 27;9:36. doi: 10.1186/s40795-023-00692-2 (PMC9969366; doi:10.1186/s40795-023-00692-2)
Supplement: Supplementary file 1 — Additional file 1: Appendix A. Nutritional Status Information Collected. Supplementary Table 1. Anthropometric Readings Collected. Supplementary Table 2. Morbidity Patterns Collected. Appendix B. Descriptive Tables. Supplementary Table 3. Descriptive Table of Food Group Consumption by Member Group. Supplementary Table 4. Descriptive Table of Sociodemographic Variable Levels. [file 40795_2023_692_MOESM1_ESM.docx]

**Appendix A: Nutritional Status Information Collected**

Supplementary Table 1: Anthropometric Readings Collected

| **Information Collected** | **How Information was Stored** |
| --- | --- |
| Member ID | Serial number was assigned to the household member along with household ID  For example, in PU-BP101/1, PU-BP101 is the household ID and 1 is the ID given to a member in the household |
| Member 1 | Adult male selected for anthropometry |
| Member 2 | Adult female selected for anthropometry |
| Member 3 | Adolescent male/female selected for anthropometry |
| Member 4 | Children selected for anthropometry |
| C1. Weight | Weight (kgs) of all the members selected for anthropometry |
| C2. Height | Height (cms) of all the members selected for anthropometry |
| C3. BMI | BMI of the all the members selected for anthropometry  BMI is calculated automatically in the Tablet-based program (weight / height^2) |
| C4. Arm circumference | Mid Upper Arm circumference (cms) of all the members selected for anthropometry |

Anthropometric information collected from individuals via enumerators is listed in the left column. The right column details how the collected information was stored in the excel dataset by detailing units or numeric values assigned to categorical information (ex. Yes or No questions).

Supplementary Table 2: Morbidity Patterns Collected

| **Information Collected** | **How Information was Stored** |
| --- | --- |
| C5A. Name and ID | The name and ID of the respondent. The enumerator has to select the name from the drop-down list that appears in the tablet. |
| C5B. Were you sick during the last week? | 1 = Yes  2 = No |
| C5C. Symptoms of sickness | If the respondent was sick, then the enumerator has to write the treatment prescribed. |
| C5D. Did you visit a doctor during the last week? | 1 = Yes  2 = No |
| C5E. Treatment received | If the respondent visited a doctor, then the enumerator has to write the treatment prescribed. |

Morbidity patterns collected from individuals via enumerators is listed in the left column. The right column details how the collected information was stored in the excel dataset by detailing units or numeric values assigned to categorical information (ex. Yes or No questions).

**Appendix B: Descriptive Tables**

Supplementary Table 3: Descriptive Table of Food Group Consumption by Member Group

|  |  | Food Group | | | | | | | | | |
| --- | --- | --- | --- | --- | --- | --- | --- | --- | --- | --- | --- |
|  |  | 1 | 2 | 3 | 4 | 5 | 6 | 7 | 8 | 9 | 10 |
| Adult male | Mean | 1 | 0.553 | 0.191 | 0.915 | 0.267 | 0.150 | 0.171 | 0.020 | 0.965 | 0.108 |
|  | SD | 0 | 0.498 | 0.393 | 0.279 | 0.443 | 0.357 | 0.377 | 0.139 | 0.184 | 0.310 |
| Adult female | Mean | 1 | 0.511 | 0.153 | 0.902 | 0.219 | 0.128 | 0.133 | 0.023 | 0.935 | 0.098 |
|  | SD | 0 | 0.498 | 0.392 | 0.241 | 0.436 | 0.371 | 0.375 | 0.149 | 0.167 | 0.341 |
| Adolescent male | Mean | 1 | 0.566 | 0.141 | 0.829 | 0.224 | 0.205 | 0.146 | 0.039 | 0.961 | 0.171 |
|  | SD | 0 | 0.497 | 0.349 | 0.377 | 0.418 | 0.405 | 0.354 | 0.194 | 0.194 | 0.377 |
| Adolescent female | Mean | 1 | 0.588 | 0.235 | 0.848 | 0.253 | 0.225 | 0.197 | 0.017 | 0.979 | 0.170 |
|  | SD | 0 | 0.493 | 0.425 | 0.360 | 0.435 | 0.418 | 0.399 | 0.131 | 0.143 | 0.376 |

The mean and standard deviation (SD) food group consumption for each member is tabulated above. A score of 1 indicates the food group was consumed, while 0 indicates the food group was not consumed. The methods section of the manuscript details the food group name that was assigned to the food group number.

Supplementary Table 4: Descriptive Table of Sociodemographic Variable Levels

|  |  |  | DDS | |
| --- | --- | --- | --- | --- |
|  | Level | Sample Size | Mean | SD |
| Marital Status | Married | 1317 | 4.277 | 1.190 |
|  | Single/never married | 497 | 4.406 | 1.330 |
|  | Widowed | 2 | 4.837 | 0.231 |
| Highest Degree of Education | No school | 6 | 4.058 | 1.081 |
|  | Primary level (classes 1-5) | 288 | 4.370 | 1.1350 |
|  | Secondary level (classes 6-10) | 943 | 4.341 | 1.256 |
|  | Technical or vocational training | 10 | 4.300 | 0.949 |
|  | Intermediate | 116 | 4.367 | 1.166 |
|  | Graduate /University (B Sc/BA/B Com/B Tech) | 92 | 4.720 | 1.207 |
|  | Postgraduate | 37 | 4.471 | 1.362 |
|  | Other | 3 | 5.000 | 0 |
| BMI Range | Underweight | 451 | 4.275 | 1.305 |
|  | Normal | 464 | 4.254 | 1.173 |
|  | Overweight | 234 | 4.294 | 1.208 |
|  | Obese | 660 | 4.399 | 1.224 |
| Type of Household Card | BPL - Below Poverty Line | 1539 | 4.298 | 1.225 |
|  | APL - Above Poverty Line | 89 | 4.676 | 1.229 |
|  | Other - No Ration Card | 188 | 4.259 | 1.254 |

The sample size, mean, and standard deviation (SD) food group consumption for levels of each sociodemographic variable analyzed is tabulated above. Table 1 of the manuscript details the level numbers assigned for each variable.
